# A pencil beam algorithm for magnetic resonance image‐guided proton therapy

**DOI:** 10.1002/mp.12854

**Published:** 2018-03-30

**Authors:** Fatima Padilla‐Cabal, Dietmar Georg, Hermann Fuchs

**Affiliations:** ^1^ Department of Radiotherapy Medical University of Vienna/AKH Vienna Austria; ^2^ Christian Doppler Laboratory for Medical Radiation Research for Radiation Oncology Medical University of Vienna Vienna Austria

**Keywords:** magnetic fields, magnetic resonance image, pencil beam algorithms, proton therapy

## Abstract

**Purpose:**

The feasibility of magnetic resonance image (MRI)‐based proton therapy is based, among several other factors, on the implementation of appropriate extensions on current dose calculation methods. This work aims to develop a pencil beam algorithm (PBA) for dose calculation of proton beams within magnetic field regions of up to 3 T.

**Methods:**

Monte Carlo (MC) simulations using the GATE 7.1/GEANT4.9.4p02 toolkit were performed to generate calibration and benchmarking data for the PBA. Dose distributions from proton beams in the clinical required energy range 60–250 MeV impinging on a 400 × 400 × 400 mm^3^ water phantom and transverse magnetic fields ranging from 0 to 3 T were considered. Energy depositions in homogeneous and heterogeneous phantoms filled with water, adipose, bone, and air were evaluated for proton energies of 80, 150, and 240 MeV, combining a trajectory calculation method and look‐up tables (LUT). A novel parametrization model, using independent tailed Gauss fitting functions, was employed to describe the nonsymmetric shape of lateral beam profiles. Integrated depth‐dose curves (IDD), lateral dose profiles, and two‐dimensional dose distributions calculated with the PBA were compared with results from MC simulations to assess the performance of the algorithm. A gamma index criterion of 2%/2 mm was used for analysis.

**Results:**

A close to perfect agreement was observed for PB‐based dose calculations in water in magnetic fields of 0.5, 1.5, and 3 T. IDD functions showed differences between the PBA and MC of less than 0.1% before the Bragg peak, and deviations of 2–8% in the distal energy falloff region. Gamma index pass rates higher than 99% and mean values lower than 0.1 were encountered for all analyzed configurations. For homogeneous phantoms, only the full bone configuration offered deviations in the Bragg peak position of up to 1.7% and overestimations of the lateral beam spot width for high‐energy protons and magnetic field intensities. An excellent agreement between PBA and MC dose calculation was also achieved using slab‐like and lateral heterogeneous phantoms, with gamma index passing rates above 98% and mean values between 0.1 and 0.2. As expected, agreement reduced for high‐energy protons and high‐intensity magnetic fields, although results remained good enough to be considered for future implementation in clinical practice.

**Conclusions:**

The proposed pencil beam algorithm for protons can accurately account for dose distortion effects induced by external magnetic fields. The application of an analytical model for dose estimation and corrections reduces the calculation times considerably, making the presented PBA a suitable candidate for integration in a treatment planning system.

## 1 Introduction

In the last years, proton beam therapy has become one of the most advanced forms of cancer treatment.^1^, ^2^ The ability to reduce radiation exposure to adjacent healthy tissue and to spare the tissue behind the tumor almost entirely makes the treatment optimal for tumors where conventional photon‐based radiotherapy results are limited.^3^, ^4^, ^5^, ^6^ Today's state‐of‐the‐art medical accelerators for proton beams are equipped with X‐ray‐based devices for image‐guided radiation therapy (IGRT), aiming to reduce geometric uncertainties during radiation delivery and to enhance the target conformity of the delivered dose.^7^, ^8^, ^9^ Limitations on the soft‐tissue contrast of images obtained with computer tomography (CT) and cone beam CT techniques, as well as the additional radiation dose to usually un‐exposed healthy tissue, stimulated research pursuing alternatives for improved imaging solutions. Magnetic resonance imaging (MRI) may be a promising candidate for real‐time image guidance in proton therapy. The superior soft‐tissue contrast and lack of exposure with ionizing radiation combined with the combination of anatomic and functional imaging are the main advantages of MRI over CT imaging.^10^, ^11^

Up to now promising results have been achieved in the development and clinical installation of hybrid MR imaging and external beam delivery units using either photon beams or gamma‐rays from Cobalt‐60 sources.^12^, ^13^ In contrary to working solutions or prototypes for MR‐guided photon beam therapy, MR‐guided proton beam therapy is still in its infancy.^14^ One fundamental question that needs to be addressed is the influence of magnetic fields on dose distribution and delivery. Previous studies based on Monte Carlo (MC) simulations showed that the lateral bending of the primary proton beam induces a shifting of the Bragg peak position, as well as a deformation of its typical shape.^15^ Further research^16^, ^17^, ^18^ showed that MRI‐proton therapy is feasible from a dosimetric point of view with the implementation of proper beam arrangement corrections. The proposed solutions were based on full MC dose calculation engines and additional compensations.

More recently, Oborn et al.^19^ simulated the delivery of proton beams into a treatment zone inside a split‐bore MRI‐guided proton therapy system. Results from these studies have shown that proton beam deflection may be predicted and corrected during planning stages. The main limitation hindering full MC‐based dose calculation models is that simulations take too long to meet clinical demand during treatment plan optimization.

For proton and carbon ion beam therapy, pencil beam (PB) algorithms represent the workhorse in clinical treatment planning systems (TPS), not only for dose calculation, but also for computerized treatment plan optimization.^20^, ^21^, ^22^ This work aims to develop a PB algorithm for protons, including a detailed description of effects induced by a transverse external magnetic field. It is designed to achieve the dose accuracy required for clinical purposes in reasonable calculation times, with the scope of a future integration into a TPS.

## 2 Materials and methods

### 2.A. PB algorithm

The proposed PB algorithm is based on a trajectory‐correction procedure and accounts for the dose deposition of a scanned proton PB using look‐up tables (LUT).^23^ The model was chosen due to its flexibility in expanding to different energies and the possibility to replace the initial calibration data with measured values. The dose at each voxel was evaluated by superimposing individual dose contributions *D*_*k*_ of single subbeams, from now and further, referred with the index “*k*.” A combination of the longitudinal depth‐dependent deposition function *E*_*dep*_(*z*) and a lateral flux probability density distribution function *f*(*x*,* y*) describing the beam broadening and lateral shifting determined the deposited energy of each beam as follows:

(1) $$D_{k}\left(x,y,z\right)=E_{\mathrm{dep}}\left(z\right)\times f\left(x,y\right)$$

#### 2.A.1. Trajectory calculation of the proton beam

The trajectory of protons in a magnetic field up to 3 T was estimated by numerically solving the relativistic Lorentz equation:

(2) $$\frac{d}{\mathrm{dt}}\left(\Gamma m_{0}\vec{v}\right)=q\left(\vec{v}\times \vec{B}\right)$$

where $\Gamma ={\left[1-{\left(\frac{|\vec{v}|}{c}\right)}^{2}\right]}^{-\frac{1}{2}}$ is the Lorentz factor of a particle with velocity $\vec{v}$, charge *q* and rest mass *m*_0_.

An iterative calculation method modified the kinetic energy of the particle at each step subtracting the corresponding energy loss of the protons according to the well‐known stopping power formula of Bethe and Bloch.^15^ At each penetration depth, using step size of 1 mm, the formula was used to determine the lateral deflection coordinates, as well as the deflection angle from the beam incidence direction.

#### 2.A.2. Longitudinal depth deposition

For every proton beam, energy deposition values in water *E*_*dep*_(*z*) at each depth alongside the beam path were stored in LUT. The interpolation between energies was based on a cubic spline method.^23^

For materials other than water, an initial equivalent depth $z_{\mathrm{eq}}^{\mathrm{init}}$ was calculated without external magnetic fields:^23^

(3) $$z_{\mathrm{eq}}^{\mathrm{init}}=z\times \frac{S_{m}\left(E,z\right)}{S_{W}\left(E,z\right)}\times \frac{\rho_{m}\left(z\right)}{\rho_{W}\left(z\right)}$$

where *ρ*_*m*_(*z*), *ρ*_*W*_(*z*) are the mass densities and *S*_*m*_(*E*,* z*) and *S*_*W*_(*E*,* z*) are the stopping power values calculated by the Bethe and Bloch formula. The mean kinetic energy of the particle was estimated iteratively by subtracting the deposited energy from the initial beam energy at each penetration depth. The subindexes *m* and *W* correspond to a desired material composition and water, respectively.

**Figure 1:**
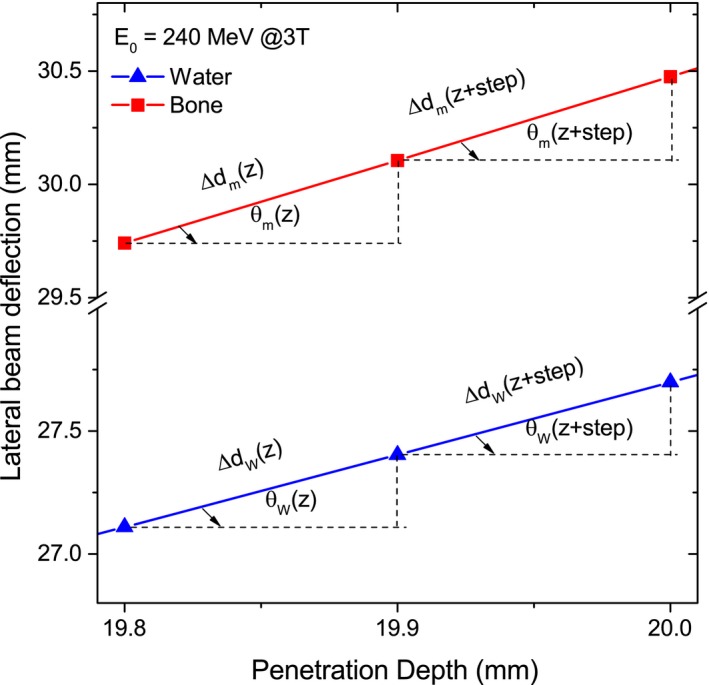
Zoom of trajectories of 240‐MeV protons in a 3‐T magnetic field region. The characteristic path lengths Δ*d*_*m*_, Δ*d*_*W*_ at two successive depth steps are shown for bone and water, respectively. [Color figure can be viewed at http://wileyonlinelibrary.com]

**Figure 2:**
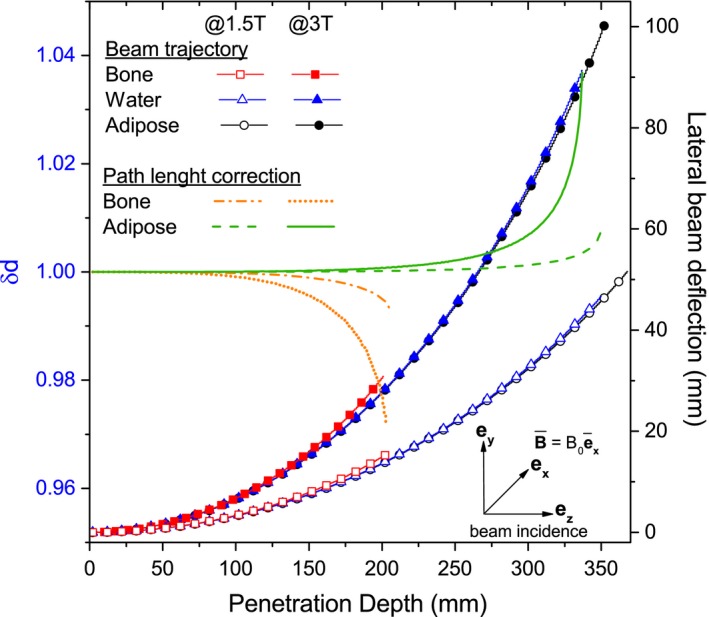
Correction factor and lateral beam deflection of 240‐MeV protons in adipose, water, and bone for 1.5‐ and 3‐T external magnetic fields. [Color figure can be viewed at http://wileyonlinelibrary.com]

In the presence of a magnetic field, due to dissimilar lateral bending of the beam in diverse materials a difference in the characteristic path lengths *δd* was also considered:

(4) $$\delta d=\frac{\Delta d_{m}\left(z\right)}{\Delta d_{W}\left(z\right)}=\frac{cos\theta_{W}\left(z\right)}{cos\theta_{m}\left(z\right)}$$

where Δ*d*_*m*_(*z*), Δ*d*_*W*_(*z*) are the path lengths of the protons and *θ*_*m*_(*z*), *θ*_*W*_(*z*) the lateral deflection angles measured from the initial beam direction at each depth step. Once more, the subindexes *m* and *W* correspond to a desired material composition and water, respectively. Typical trajectories of protons in water and bone are exemplified in Fig. 1. Fig. 2. shows the calculated proton trajectories for different materials and the correction factor *δd* for high‐energetic 240‐MeV protons at 1.5‐ and 3‐T fields, respectively. The water‐equivalent depth *z*_*eq*_ was finally determined combining both scaling factors as follows:

(5) $$z_{\mathrm{eq}}=z_{\mathrm{eq}}^{\mathrm{init}}\times \delta d$$

#### 2.A.3. Lateral depth deposition

**Figure 3:**
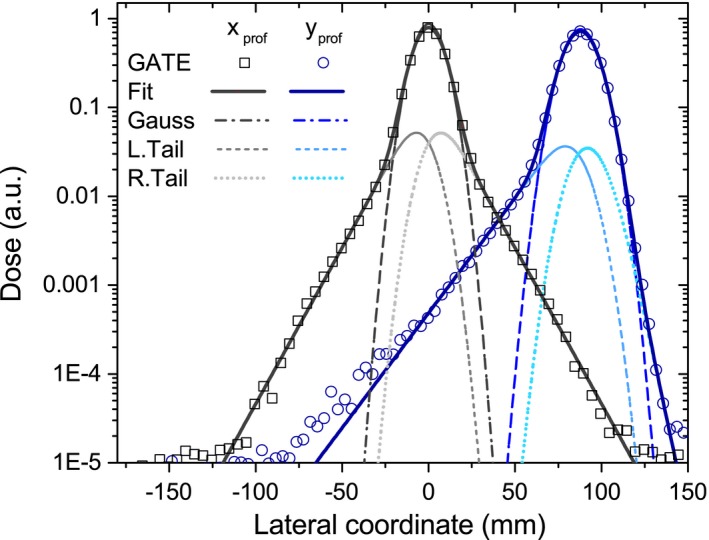
Fitting function employed for transverse dose profiles scored at 320 mm depth in water for the parallel (squares) and perpendicular (circles) coordinate referred to the magnetic field orientation. [Color figure can be viewed at http://wileyonlinelibrary.com]

Lateral integrated dose profiles in water were carefully analyzed to evaluate the effect of the magnetic field on the shape and broadening of the proton beam. Shifted and nonsymmetric dose profiles were observed for the coordinate transverse to the magnetic field orientation, always referred to as *y*. In contrast, lateral profiles for the coordinate parallel to the magnetic field orientation, denoted as *x*, showed only the well‐known broadening effect due to nuclear and Multiple Coulomb scattering interactions. An exact analytical solution, describing both *x* and *y* profiles, seemed to be challenging. Instead, a parametrization procedure, based on the direct fitting of the MC dose lateral profiles, was proposed. The primary Gaussian beam shape was tailed with two independent exponential functions. The central area described the primary interactions of the beam, while the tailing components included the lateral distortions due to multiple Coulomb scattering, nuclear interactions, and charge drifting. At each penetration depth, the intensity *f*(*x*) of the peak was estimated as a 7 free parameters function:

(6) $$\begin{array}{cc} f(x)= & \;A\times [\left(1-f_{L}-f_{R}\right)\times G\left(x\right)+f_{L}\\ & \times LTail\left(x\right)+f_{R}\times RTail\left(x\right)] \end{array}$$

$$G\left(x\right)=\frac{1}{\sqrt{2\pi }\sigma }exp\left[\frac{{\left(x-x_{m}\right)}^{2}}{2\sigma^{2}}\right]$$

$$\begin{array}{cc} & LTail(x)=\\ & \frac{1}{2\gamma_{L}\sigma exp\left(-\frac{1}{2\gamma_{L}^{2}}\right)}exp\left[\frac{\left(x-x_{m}\right)}{\gamma_{L}\sigma }\right]erfc\left[\frac{\left(x-x_{m}\right)}{\sqrt{2}\sigma }+\frac{1}{\sqrt{2}\gamma_{L}}\right] \end{array}$$

$$\begin{array}{cc} & RTail(x)=\\ & \frac{1}{2\gamma_{R}\sigma exp\left(-\frac{1}{2\gamma_{R}^{2}}\right)}exp\left[\frac{(x_{m}-x)}{\gamma_{R}\sigma }\right]erfc\left[\frac{\left(x_{m}-x\right)}{\sqrt{2}\sigma }+\frac{1}{\sqrt{2}\gamma_{R}}\right] \end{array}$$

where *f*_*L*_, *f*_*R*_ represent the fractional contribution to the total peak area (*A*) of the left and right tailing functions, *x*_*m*_, *σ* the mean and the corresponding standard deviation values of the Gaussian central beam, and *γ*_*L*_, *γ*_*R*_ are the rates of left and right exponential components. Fig. 3 shows two transverse dose profiles and the corresponding fitted functions for a 240‐MeV proton beam in water.

Data for the lateral beam were obtained from simulations of the two‐dimensional XZ and YZ projected dose distributions in water. The total integrated area, as well as the other six parameters, was tabulated for each penetration depth into two corresponding files for the parallel and perpendicular coordinates corresponding to the magnetic field orientation.

#### 2.A.4. Beam splitting techniques

Beam decomposition into smaller subbeams is a common technique to describe the changes in range and lateral profiles caused by heterogeneities adjacent to the beam axis.^20^, ^24^, ^25^, ^26^ The number of subbeams *k* employed for calculations varied according to the phantom type used for benchmarking. The beam splitting optimization procedure proposed by Fuchs et al 23 was employed through all our calculations. Moreover, a dynamic shifting of the beam central position was included. The beam center location was corrected at each penetration depth according to its lateral bending inside the material. Subspots were equidistantly distributed to a lateral distance of three times the initial beam sigma *σ*_*x*_, *σ*_*y*_.

#### 2.A.5. Total dose calculation

The final 3D dose distribution was achieved by superimposing the contribution of each subbeam *k*.

(7) $$\begin{array}{cc} D(x,y,z)= & \;\frac{1}{m\left(x,y,z\right)}\sum_{k}E_{k-dep}^{\mathrm{LUT}}\left(z_{\mathrm{eq}}\right)\\ & \times f_{x}^{\mathrm{nucLUTX}}\left(x,z_{\mathrm{eq}}\right)\times f_{y}^{\mathrm{nucLUTY}}\left(y,z_{\mathrm{eq}}\right) \end{array}$$

where $m\left(x,y,z\right)$ is the mass of each analyzed voxel. The dose contribution of each subbeam *k* was assessed at water‐equivalent depths, multiplying the total integral dose distribution $E_{\mathrm{dep}}^{\mathrm{LUT}}\left(z_{\mathrm{eq}}\right)$ values and the evaluated probability density functions $f_{x,y}^{nucLUTx,y}\left(x,y,z_{\mathrm{eq}}\right)$. Typical dose volume grids of 1 × 1 × 1 mm^3^ were used throughout this paper to compare with benchmark data from MC simulations.

#### 2.A.6. Software implementation

The PB algorithm was implemented in C++ using the ROOT framework (version 5.34) based on a nonfield algorithm from our research team.^23^, ^27^ ROOT is a scientific software framework developed at CERN used for big data processing, statistical analysis, visualization, and storage.^28^ Data analysis and fitting procedures were performed using C++/Root packages and the commercial software MATLAB R2016b, The MathWorks, Inc., Natick, Massachusetts, United States.

### 2.B. Data generation and validation

Lack of dosimetric experimental data for proton beams in magnetic fields, implied that the best current possible tool to benchmark our semi‐analytical calculations, was to use a previously validated GATE toolkit for proton therapy applications.^29^, ^30^, ^31^, ^32^ Monte Carlo (MC) simulations using GATE v7.1 alongside GEANT4.9.4p02 were performed to calculate the input and benchmark data for the algorithm. GATE is a free and general‐purpose MC toolkit, based on the GEANT4 simulation platform, developed by the OpenGATE collaboration since 2001.^33^

Parallel and monoenergetic proton beams, covering energies from 40 to 250 MeV in 10‐MeV intervals, with a typical spot size of *σ*_*x*_, *σ*_*y*_ = 3 mm impacting on a 400 × 400 × 400 mm^3^ water phantom were used as reference. The entire system was placed within a homogeneous magnetic field, oriented in the positive *x* direction and transverse to the beam direction, arbitrarily selected alongside the *z* axis. The input data for the algorithm were generated from the energy deposition in water of the above‐mentioned 22 monoenergetic proton beams interacting with 0.5‐, 1.5‐, and 3‐T external fields. The dose acquisition grid was set to 400 × 400 × 0.1 mm^3^ for the determination of integral depth‐dose (IDD) functions, and to 1 × 1 × 1 mm^3^ for the estimation of tridimensional dose maps. The electromagnetic and hadronic processes were implemented using the physic list QGSP_BERT_EMV according to recommendations from the OpenGATE collaboration (the MA‐Physics‐list‐protons.mac physics list). Production cuts of 0.1 mm and 1 mm were selected for particle transport inside the phantoms and the rest of the geometry, respectively. The number of particles per simulation was set to 10^7^ to ensure statistical errors below 1% in our calculations. The ionization potentials of air, water, bone, and adipose tissues employed 85.7, 75, 91.9, and 63.2 eV, respectively, accordingly to the NIST/PSTAR database.^34^

All simulations were conducted on the in‐house computing cluster from the MOCCAMED group. The system combines one central node and more than 14 workstations throughput the batch processing system Condor.

The accuracy and parameter range of validity of the proposed algorithm was analyzed by means of direct comparison with results from GATE simulations in homogeneous and heterogeneous cubic 400 × 400 × 400 mm^3^ phantoms. For the homogeneous configuration, phantoms were filled with water, adipose, or bone tissue. Afterward, a sandwich configuration was used alternating 20‐mm‐thick slabs of water with adipose, bone, and air at the entrance region to rate the effect of boundaries and identify critical regions of the algorithm. The last configuration was designed to consider heterogeneities parallel to the beam axis. Two opposing slabs (bone and air) of 20 mm thickness were inserted at 20 mm depth in water.

#### 2.B.1. Evaluation criteria

Energy deposition maps were obtained for proton energies of 80, 150, and 240 MeV in magnetic field regions of 0.5 T, 1.5 T, and 3 T. The computed integral depth‐dose deposition functions, lateral profiles, and bidimensional dose maps were obtained from 3D energy deposition maps using a dose acquisition grid of 1 × 1 × 1 mm^3^.

The longitudinal shifting of the total beam range determined at 80% of the distal dose level (ΔR_80_), as well as the local dose differences at each depth, was selected as parameters for the evaluation of the integral depth‐dose distributions. Mean values of dose differences Δ*D*_*mean*_ were calculated from the weighted average of the histogram distribution to smooth the influence of the falloff region in the overall result. For the average calculation, only dose values higher than 1% of the maximal dose were considered. Lateral beam profiles at 20%, 50%, and 80% of the total beam range were compared to evaluate the precision of the proposed model. The central position and the full width at half maximum (FWHM) of the Gaussian component of the beam was determined using the PBA and balanced with the values predicted by GATE. Hereinafter, these two parameters will be referred in the text as the lateral bending coordinate and beam spot size. Only for the opposing slabs phantom, lateral beam profiles were scored at 30 mm, 50%, and 80% of the total beam range, to ensure that the first lateral profile was scored in the inhomogeneous region of materials. Finally, two‐dimensional energy deposition maps were compared by means of the *γ* ‐index method, following a 2%/2 mm criteria of the global maximum. Gamma indices were calculated at every single voxel, where the received dose was higher than 0.1% of the maximal dose D_max_. The mean parameter value, as well as the *γ* ‐index passing rates, was computed for all analyzed configurations.

## 3 Results

### 3.A. Homogeneous media

**Figure 4:**
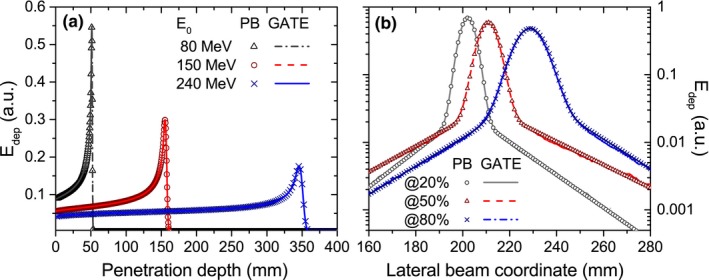
Calculated (lines) and simulated (markers) IDD functions of 80‐, 150‐, and 240‐MeV protons (a) and lateral beam profiles of a 240‐MeV proton beam scored at 20%, 50%, and 80% of the total beam range (b) in a homogeneous water phantom placed in a 1.5‐T transverse magnetic field region. [Color figure can be viewed at http://wileyonlinelibrary.com]

Integral depth‐dose deposition functions and lateral beam profiles in water exposed to a 1.5‐T magnetic field are shown in Fig. 4. For water, an excellent agreement was obtained. Ranges predicted with the PBA are identical to the ones calculated with GATE and encountered local dose differences were less than 0.1%.

Table 1. summarizes the precision on range estimation and mean local dose divergences for diverse materials, beam energies, and magnetic field intensities. Doses exhibited a mean difference between 0.1% and 3.6% compared with MC values.

**Table 1:** IDD evaluation of all tested homogeneous phantom configurations. Range shifting and mean dose difference are presented for the 0.5‐, 1.5‐, and 3‐T external magnetic fields

		0.5 T		1.5 T		3 T	
Material	E_0_	ΔR_80_ (mm; %)	Δ*D*_mean_ (%)	ΔR_80_ (mm; %)	Δ*D*_mean_ (%)	ΔR_80_ (mm; %)	Δ*D*_mean_ (%)
Water	80	–	<0.1	–	<0.1	–	<0.1
	150	–	<0.1	–	<0.1	–	<0.1
	240	–	<0.1	–	<0.1	–	<0.1
Adipose	80	–	−0.5	–	−0.7	0.1; 0.2	−1.1
	150	–	−0.8	–	−0.6	0.1; 0.1	−0.5
	240	–	−0.4	0.3; 0.1	−0.9	0.5; 0.1	−0.7
Bone	80	0.5; 1.7	−1.8	0.4; 1.3	−2.0	0.3; 1.0	−1.7
	150	0.4; 0.4	−0.2	0.2; 0.2	−1.0	0.5; 0.6	−1.5
	240	0.5; 0.2	0.3	2.0; 1.0	0.7	6.7; 3.5	3.5

Differences in the bending coordinate and beam spot size calculation of a 240‐MeV proton beam are shown in Table 2. The lateral beam deflection predicted with the PBA agreed very well with simulated results, showing a maximal deviation of 0.6 mm. The beam broadening is very well described for water and adipose tissue, but an overestimation of the profile width closer to the Bragg peak region for bone is present. Results for lower energy proton beams (80, 150 MeV) showed a similar or mostly better agreement compared with the 240‐MeV protons.

**Table 2:** Comparison of 240‐MeV proton transverse beam profiles simulated to PBA scored at three different penetration depths in water, adipose, and bone. The differences in lateral shifting (Δy) and spot size (ΔW) between PBA calculations and GATE results are given for 0.5‐, 1.5‐, and 3‐T external magnetic fields

	0.5 T		1.5 T		3 T	
Material	Δy (mm)	Δ*W* (mm)	Δy (mm)	Δ*W* (mm)	Δy (mm)	Δ*W* (mm)
Water						
20% BP	<0.1	<0.1	<0.1	<0.1	−0.1	<0.1
50% BP	<0.1	<0.1	−0.2	−0.2	−0.3	<0.1
80% BP	<0.1	−0.2	−0.3	−0.3	−0.5	−0.2
Adipose						
20% BP	<0.1	<0.1	−0.1	−0.1	−0.1	<0.1
50% BP	<0.1	−0.3	−0.2	−0.3	−0.3	−0.2
80% BP	<0.1	−0.6	−0.3	−0.7	−0.6	−0.5
Bone						
20% BP	<0.1	<0.1	<0.1	−0.1	<0.1	<0.1
50% BP	<0.1	−0.8	<0.1	−0.9	−0.2	−0.9
80% BP	<0.1	−2.8	<0.1	−3.9	−0.4	−3.6

### 3.B. Heterogeneous media

#### 3.B.1. Slab configuration

**Figure 5:**
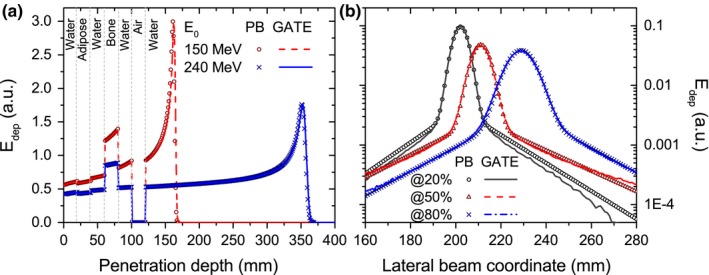
Proton IDD functions (a) for 150‐MeV and 240‐MeV protons and transverse profiles (b) for a 240‐MeV beam impinging a slab‐like inhomogeneous phantom located in a 1.5‐T magnetic field region. The profile closer to the beam entrance at 20% (circles) was sliced inside the bone slab, showing the PBA results a wider profile than MC simulations in the halo region. For reference, dashed lines were added to indicate the geometrical position and material composition of the inhomogeneities. [Color figure can be viewed at http://wileyonlinelibrary.com]

**Figure 6:**
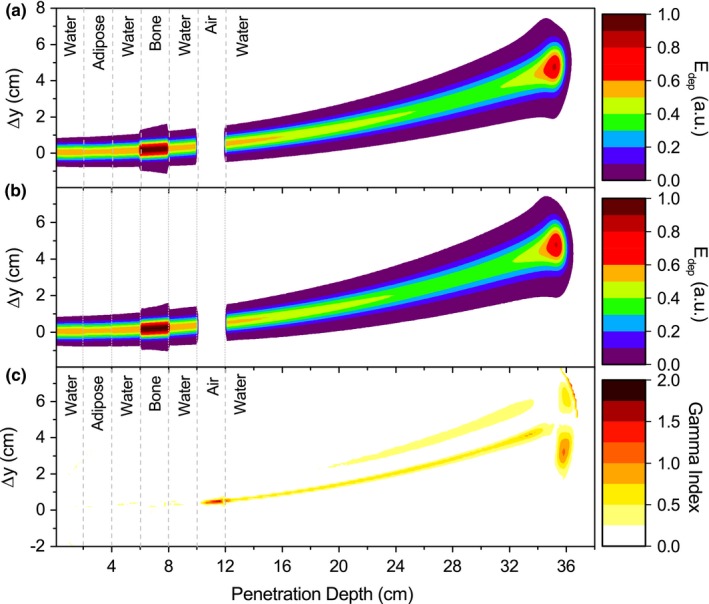
Energy deposition maps calculated with the PBA (a) and GATE (b) and gamma index map (c) for a 240‐MeV proton beam and 1.5‐T field in a slab‐like heterogeneous phantom. Dashed lines were added to indicate the slab locations inside the phantom. [Color figure can be viewed at http://wileyonlinelibrary.com]

Depth‐dose and lateral profiles in a slab phantom configuration are compared in Fig. 5. Maximal fluctuations in range estimation of 0.6% were encountered for all analyzed cases, while maximum mean discrepancies on dose calculation for 150‐ and 240‐MeV protons exposed to a 3‐T field were 0.6% and 0.3%, respectively. Local dose differences below 1%, 2%, and 15% were observed in the water interface with adipose, bone, and air material, respectively. Lateral bending and the characteristic spot sizes agreed within 0.2 mm for both energies and all three field intensities. Two‐dimensional dose distributions resulting from PBA calculations and MC simulations are also shown in Fig. 6 together with the *γ* ‐index analysis map. The only considerable deviations were encountered in air and water in the Bragg peak falloff region. The effect on the tilting of the MC dose distributions was described previously by Fuchs et al.^15^ and observed at the end of the beam trajectory also for the PBA calculations. In the Bragg peak region, agreement between results is excellent, showing minor deviations only in the dose regions lower than 5% of the peak maximum dose.

#### 3.B.2. Lateral configuration

**Figure 7:**
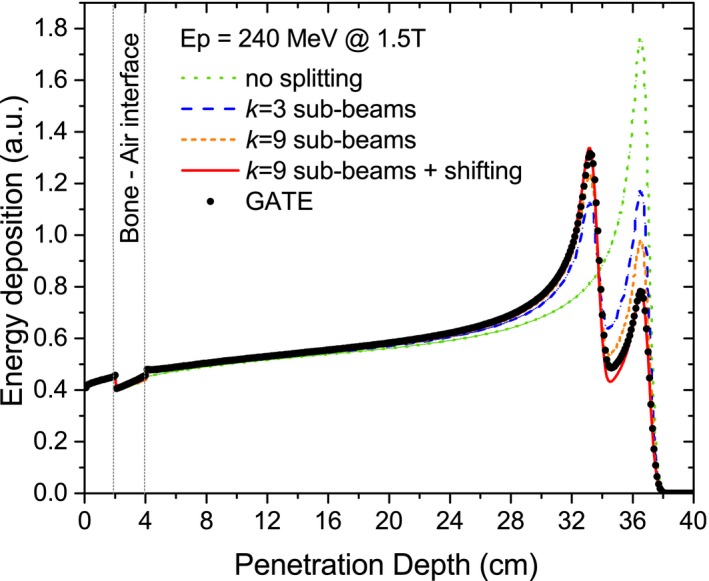
IDD functions simulated with GATE and calculated by the PBA combining an initial beam splitting technique and a central beam shifting for 240‐MeV protons at 1.5‐T field in a lateral slab phantom configuration. [Color figure can be viewed at http://wileyonlinelibrary.com]

As expected, the agreement of the proposed PBA decreased when heterogeneities were located parallel to the beam direction. Different numbers of subbeams *k* were tested for the last configuration, starting from nonsplitting (*k* = 1) to a high number (*k* = 15). Fig. 7 shows that a progressively better level of agreement was obtained by combining a higher number of subbeams with an adequate central beam shifting.

**Figure 8:**
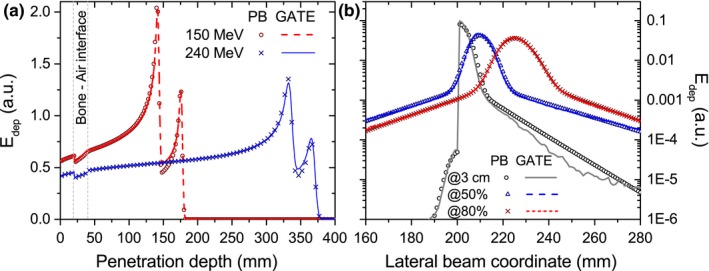
IDD functions from 150‐ and 240‐MeV protons (a) and transverse profiles resulting from interaction of 240‐MeV protons (b) impinging the opposing material slab phantom located in a 1.5‐T magnetic field region. PBA calculations were performed using *k* = 9 subbeams and a shifting of the beam initial position. The profile closer to the beam entrance (circles) was scored in air, showing an acceptable performance of the PBA also in the low‐dose region. Dashed lines indicate the opposing material slab inside the phantom. [Color figure can be viewed at http://wileyonlinelibrary.com]

The initial beam splitting combined with the corresponding lateral correction of the initial subbeams positions was sufficient to obtain close results between the PBA and GATE (Fig. 8) for the integral depth‐dose and lateral profiles. The inaccuracy in the range determination never exceeded 0.7%; meanwhile, the mean dose deviations were lower than 2.1% and 1.3% for the 150‐ and 240‐MeV beams, respectively. Similar to the slab phantom configuration, the lateral bending and the characteristic spot size agreed within 0.2 mm for the two energies and three field intensities.

**Figure 9:**
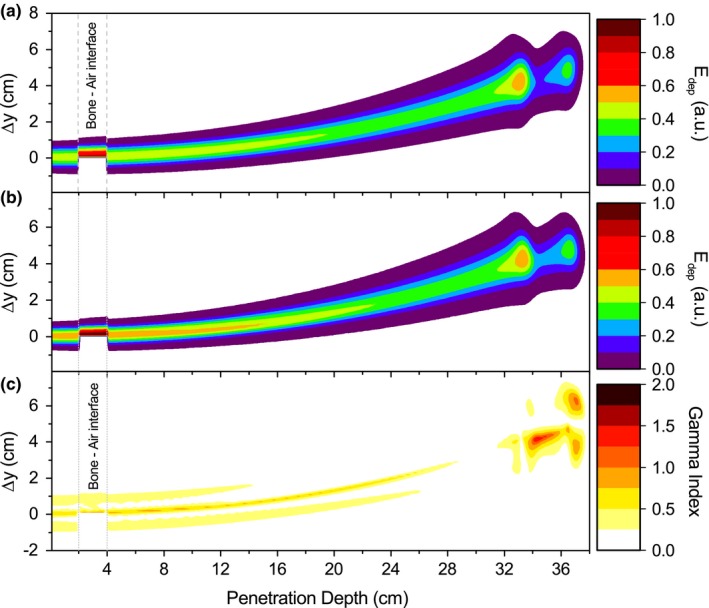
Energy deposition maps calculated with the PBA (a) and GATE (b) and gamma map index (c) for a 240‐ MeV proton beam and 1.5‐T field in a lateral heterogeneous phantom. A nonmatching region between the two characteristic Bragg peaks is observed from the gamma index plot. Dashed lines indicate the slab location inside the phantom. [Color figure can be viewed at http://wileyonlinelibrary.com]

The comparison between the Bragg peak regions obtained from PBA calculations and MC simulations (Fig. 9) showed inaccuracies up to 54% between, resulting in gamma indexes values above 1 and a decrease on the gamma index pass rates. An overview of the results of gamma index mean values *γ*_mean_ and pass rates *γ* < 1 from all the analyzed phantom configurations is outlined in Table 3. Even for these challenging geometries, the algorithm showed an overall good performance for all analyzed configurations.

**Table 3:** Gamma index evaluation for all the analyzed configurations (homogeneous + heterogeneous phantoms). The mean values *γ*_mean_ and the index passing rates *γ* < 1 are reported for 0.5‐, 1.5‐, and 3‐T fields

		0.5 T		1.5 T		3 T	
Material	E_0_	*γ*_mean_	*γ* < 1 (%)	*γ*_mean_	*γ* < 1 (%)	*γ*_mean_	*γ* < 1 (%)
Water	80	<0.1	100	<0.1	99.3	<0.1	99.9
	150	<0.1	100	<0.1	99.9	<0.1	99.9
	240	<0.1	100	<0.1	99.9	<0.1	99.9
Adipose	80	0.1	100	0.1	100		99.9
	150	<0.1	100	<0.1	100		99.9
	240	<0.1	99.5	<0.1	99.4		99.4
Bone	80	0.1	100	0.2	96.3	0.2	99.0
	150	0.3	94.9	0.5	91.4	0.3	92.3
	240	0.3	92.8	0.4	85.4	0.5	84.4
Slab	150	0.2	100	0.2	99.2	0.2	98.4
	240	<0.1	99.9	0.1	99.5	0.2	98.8
Lateral	150	0.1	99.4	0.1	99.3	0.2	98.5
	240	<0.1	99.7	<0.1	99.8	0.2	98.9

Even though only data for a magnetic field of 1.5 T is shown on the graphs, the PBA was validated also for field values of 0, 0.5, 1.5, and 3 T. The performance of the algorithm was comparable to the results presented above.

## 4 Discussion

An accurate dose calculation method in the presence of external magnetic fields is essential for MRI‐based proton therapy. Although Monte Carlo methods represent the most precise tool for dose calculation, it's well known that nowadays a clinical implementation demands a substantial amount of computational time and resources. Up to now, in most of particle therapy treatment planning systems, dose calculation and plan optimization algorithms are based on pencil beam kernels. A proper modification of this algorithms predicting and correcting the proton beam deflection within magnetic fields represent a viable option for compensate the encountered dose distortions. Besides the discussed model in this work for particle trajectory estimation, another alternative method, Raytracing Algorithm for Magnetic Deflection of Ions in Media (RAMDIM), was presented recently.^35^ In this case, correction parameters Δ*γ* and ΔE_0_ for the initial beams were proposed for different proton energies and magnetic flux densities to compensates the Bragg peak retraction and the lateral beam bending. The new proposed PBA offers a fast and accurate alternative for dose calculation. So far, the study was conducted using monoenergetic proton beams and homogenous magnetic fields covering the range of typical MRI systems and a high‐field extreme condition. Moreover, the trajectory estimation method showed high versatility to account for changing magnetic fields and heterogeneous tissues composition not affecting the performance of the algorithm. The next logical step after this work is to recalculate the LUT for a realistic beam model and magnetic field maps, including also beam energy spread and fringe fields. A future implementation including complex patient geometries is foreseen by the authors to complete the benchmarking. This is current work in progress and a project on its own.

To assess the speed of the PBA, calculations were performed on a PC with 8 GB RAM and a 64‐bit dual‐core processor Intel^®^ Core™ i7‐4510U at 2.00 GHz using typical calculations grids of 1 × 1 × 1 mm3 in 400 × 400 × 400 voxels. Independently of phantom configuration and for the applied maximum proton energy, the algorithm processed each single beam in less than 0.1 s. When beam splitting techniques are required, the calculation times increase by a factor between 4 and 25 times for 9 and 81 subbeams, respectively. Within the scope of this work, no speed optimization techniques were studied. However, for a further implementation in an adaptive setting, a faster performance should be possible.

In PB algorithms for protons, like the ones proposed in Fujimoto et al^22^ and da Silva et al,^25^ the central beam broadening due to multiple Coulomb scattering effects and nuclear interactions is described using single or multiple Gaussian beams. More complex parametrization models, based on direct fitting of lateral dose deposition functions,^36^ are very common to account for the low‐dose halo region. Although these models showed to be capable of an accurate dose distribution description, the extension to the magnetic field case is not straightforward. Due to the symmetric distribution of the above‐mentioned fitting functions, they are not capable to describe the characteristic deformations of the lateral profiles encountered in the presence of magnetic fields as depicted in Fig. 3. The novel parametrization model allows an accurate and simultaneous description for both symmetrically and nonsymmetrically distorted lateral dose profiles. The flexibility of the algorithm permits its use also in regions where no magnetic field is applied. However, one disadvantage compared with the single or double Gaussian models is the higher number of parameters and complexity.

For all analyzed lateral profiles, the fractional contributions of the Gaussian central beam and the two tailings of the total peak area depend on the proton energy and magnetic field intensity. Deformed peak shapes are predicted within the model with different contributions from the rising and falling peak regions. The strong dependence on the tailing components in our parametrization model shows the necessity to account not only for central but also for distant contributions of the beam. During our calculations, lateral energies depositions as far as 40 times the initial beam sigma were considered. For high‐energy protons and magnetic field intensities, analysis on a narrower lateral region can deteriorate the precision on dose estimation in the Bragg peak region from values lower than 0.1% to 6%. For low‐energy beams and magnetic field intensities, this effect is negligible. The performance of the algorithm was also tested in the absence of a magnetic field, and results showed similar accuracy compared with the low‐intensity field cases.

A close to perfect agreement of the integral dose distribution for all proton energies and homogeneous magnetic field intensities in water was reached. Significant deviations were encountered in bone and air materials and in boundary regions for the slab phantoms. An underestimation of the dose calculated with the PBA at the water–air interface was observed for different proton beam energies. It is well known that PBA are not capable to describe the surface doses in the proximal and distal side of air cavities due to the electron return effect. Even when this effect for proton beams is lower than for external photon beam therapy,^15^ a more detailed study on the effect of air cavities on the performance of the algorithm is envisaged.

For materials other than water, the water‐equivalent depth scaling reproduced accurately the IDD functions for all the analyzed configurations, except for the full homogeneous bone phantom. However, a systematic overestimation of the beam broadening was observed in the low‐dose halo region for bone, showing maximum deviations for high proton energies and highest magnetic field intensity. As expected, the implemented approximations on water‐equivalent path‐length conversion and the direct interpolation of the beam parameters from water failed to describe extreme conditions on material densities and boundaries. Instead of a direct interpolation model, an analytical formula describing a material‐dependent scattering length based on the Fermi‐Eyges theory^37^ is more desirable to improve accuracy. However, the implementation on these complex analytical methods increases considerably the calculation times. Considering that the accuracy of the PBA was only reduced in the presence of unrealistic clinically relevant cases during evaluation and overall accurate results were obtained for “realistic scenarios” in terms of technical realization (0.5 T, 1.0 T, and 1.5 T), the extension of the PBA in this respect can be also questioned.

For phantoms with heterogeneities located parallel to the beam direction, discrepancies in dose calculations between the PBA and Monte Carlo simulations also increased. A potential weakness of PBA is the difficulty to describe absolute dose values within the Bragg peak regions when heterogeneities are placed transverse to the beam propagation. This intrinsic limitation of PBA can be corrected using widespread splitting techniques.^22^, ^23^, ^24^ This approach is still valid to describe superficial heterogeneities, but fails to foresee heterogeneities located deep inside the media when magnetic fields are applied. The greater the beam deflection at the depth where heterogeneities are inserted, the lower the accuracy of the PBA. In contrast to the nonfield case, for bended proton trajectories, the material interface would then not be perceived by the central beam spot, instead only by a fraction of the lateral area. Therefore, besides the initial beam splitting, a shifting of the central position was required. Although the implemented corrections improved the accuracy of the results, the straight paths of protons considered during beam transport as well as the symmetric distribution of subbeams during splitting are approximations to actual particle paths inside the media. When high lateral bending and distortions of the beam lateral spots are encountered, a combination using a higher number of subbeams is required to improve the precision of the calculations. In the scope of this work, excessive number of subbeams was tested to analyze the strength and limitations of the algorithm. For clinical applications, a compromise balancing the desired accuracy and speed is required.

The flexibility of the presented PBA in implementation into specific configurations, using for example adapted magnetic field maps and/or experimental measurements for longitudinal and lateral dose distributions, combined with the considerable reduction on calculation times, makes it a suitable candidate for further integration in a treatment planning system.

## 5 Conclusions

The presented algorithm combines a numerical trajectory calculation of proton beams in different media and external magnetic fields with a parametrization model based on Monte Carlo simulations in water, including cases where no magnetic field is applied. The proposed model offers a fast and suitable alternative for MC‐based dose calculations. As the next stage, it is envisaged to perform the experimental verification of magnetic fields effects on dose distributions from proton beams.

## Conflict of interest

The authors have no conflict of interest to report.
